# Modified Retzius-sparing robot-assisted radical prostatectomy for cases with anterior tumor: a propensity score-matched analysis

**DOI:** 10.1007/s00345-024-04807-7

**Published:** 2024-03-20

**Authors:** Jiajun Qian, Yao Fu, Giancarlo Marra, Feifei Zhang, Xiao Wu, Danyan Li, Linfeng Xu, Xuefeng Qiu, Weidong Gan, Hongqian Guo

**Affiliations:** 1 Department of Urology, Nanjing Drum Tower Hospital Clinical College of Nanjing Medical University, Nanjing, 211166 China; 2 Department of Urology, Affiliated Drum Tower Hospital, Medical School of Nanjing University, Nanjing, 210008 China; 3 Institute of Urology, Nanjing University, Nanjing, 210008 China; 4 Department of Pathology, Affiliated Drum Tower Hospital, Medical School of Nanjing University, Nanjing, 210008 China; 5 Department of Urology, San Giovanni Battista Hospital, Città della Salute e della Scienza and University of Turin, Turin, Italy; 6 Department of Anesthesiology, Affiliated Drum Tower Hospital, Medical School of Nanjing University, Nanjing, 210008 China; 7 Department of Radiology, Affiliated Drum Tower Hospital, Medical School of Nanjing University, Nanjing, 210008 China

**Keywords:** Modified, Retzius-sparing, Urinary continence, Positive surgical margin, Anterior prostate cancer

## Abstract

**Objective:**

To compare the outcomes between a modified Retzius-sparing robot-assisted radical prostatectomy (mRS-RARP) technique and conventional robot-assisted radical prostatectomy (Con-RARP) technique for cases with anterior prostate cancer (PCa), especially positive surgical margin (PSM) rates and urinary continence (UC).

**Patients and methods:**

We retrospectively included 193 mRS-RARP and 473 Con-RARP consecutively performed by a single surgeon for anterior PCa. Perioperative complications, pathology, and continence were compared after propensity score matching using 9 variables.

**Results:**

After matching (*n* = 193 per group), PSM were not significantly different in the two groups (16.1% in mRS-RARP group vs. 15.0% in Con-RARP group, *p* = 0.779). The UC at catheter removal and at 1-month was significantly higher in the mRS-RARP (24.9% vs. 9.8%, *p* < 0.001; 29.0% vs. 13.5%, *p* < 0.001, respectively), but not at 3-, 6-, and 12-month follow-ups (*p* = 0.261, 0.832, and 0.683, respectively).

**Conclusion:**

mRS-RARP seems to be an oncologically safe approach for patients with anterior PCa. Compared with the conventional approach, mRS-RARP approach shows benefits in the short-term postoperative UC recovery.

**Supplementary Information:**

The online version contains supplementary material available at 10.1007/s00345-024-04807-7.

## Introduction

Radical prostatectomy (RP) is one of the reference standards for the treatment of localized prostate cancer (PCa) [1, 2]. With the development of surgical robots, robot-assisted RP (RARP) has been widely adopted due to its optical magnification, three-dimensional vision, and instruments with 7 degrees of freedom. Conventional RARP (Con-RARP) is performed using the anterior approach including the extraperitoneal and transperitoneal approaches. Urinary continence (UC), one of the most important indicators of quality of life after RP, closely relates to the treatment satisfaction in prostate cancer survivors [3]. With the development of surgical instruments and techniques [4], more than 80% of patients could regain UC 12 months after Con-RARP [5]. However, there are still ~ 50% patients suffering from the usage of pads early after Con-RARP [5].

In 2010, Retzius-sparing robot-assisted RP (RS-RARP) was initially described by Galfano et al. [6]. Compared to the conventional approach, a better early postoperative UC recovery in patients underwent RS-RARP was reported by several randomized controlled trials (RCTs) [7–9] and meta-analyses [10–12]. Despite the better functional recovery, Retzius-sparing approach was reported to be significantly associated with the increased risk of positive surgical margin (PSM) rate [10, 13], leading to a potential concern regarding the cancer control of this approach. PSM rate might be higher in case of anteriorly located PCa after RS-RARP [14].

To overcome this potential disadvantage, we developed a modified Retzius-sparing technique, allowing an easier access to lateral and anterior prostatic anatomy. Hence, we aimed to describe the surgical steps of this modified RS-RARP (mRS-RARP) and to assess its pathological, oncological and functional results in comparison to the conventional approach of RARP (Con-RARP).

## Patients and methods

### Patients and study design

We retrospectively included 193 consecutive mRS-RARP (July 2018 and September 2021) and 473 consecutive conventional RARP (June 2014–September 2016) performed for localized or locally advanced anterior tumors contacting with the anterior capsular of the prostate on preoperative magnetic resonance imaging (MRI) (Supplementary Fig. 1). All surgeries were carried out by a single operator (H.G.) who started performing a Retzius-sparing technique from January 2017 (with the exception of prostate sizes > 100 cc). Men with neoadjuvant hormone therapy or early adjuvant radiotherapy or hormone therapy were excluded. A propensity score matching was used to balance baseline information including age, BMI, PSA, prostate volume, ASA score, risk stratification, biopsy ISUP group, clinical stage between the two groups (Supplementary Table 1). Finally, 193 patients with anterior tumors who underwent Con-RARP were included for analysis. The baseline information, pathological features, perioperative, oncological, and UC outcomes between two approaches were compared. We retrospectively consulted medical history of enrolled patients and judged complications by progress note and medical advice. Complications were graded by Clavien–Dindo classification. Since more than half of the included patients had moderate-to-severe erectile dysfunction preoperatively, we did not assess the potency outcomes. All procedures performed in this study were in accordance with the Declaration of Helsinki and approved by the Ethics Committee of the Drum Tower Hospital, Medical School of Nanjing University.

### Surgical techniques

**Fig. 1:**
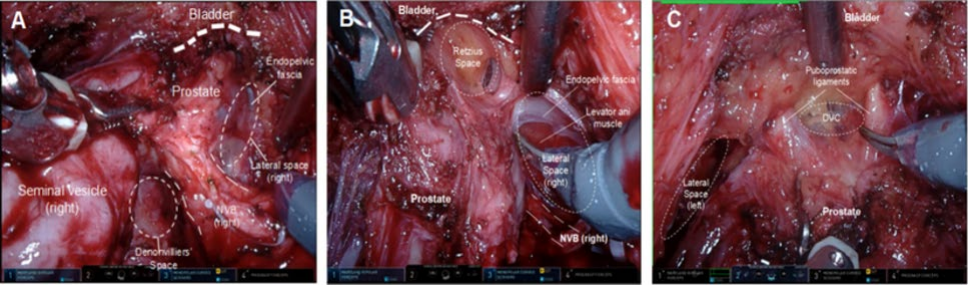
Dissection of the posterolateral (**A**), anterolateral (**B**), and anterior (**C**) part of prostate during the modified Retzius-sparing approach of robot-assisted radical prostatectomy. *VD* vas deferens, *SV* seminal vesicles, *P* prostate, *DF* seminal vesicles, *PF* prostatic fascia, *EPF* endopelvic fascia, *NVB* neurovascular bundle, *B* bladder, *DVC* dorsal venous complex

All surgical procedures were performed using a 4-arm da Vinci Surgical System (Intuitive Surgical, Sunnyvale, CA, USA). The Con-RARP approach was performed using the Patel technique, through a transperitoneal, antegrade approach. The details of surgical techniques had been stated previously [15]. The mRS-RARP, performed via the transperitoneal approach, was similar to that described by Egan et al. [16] and Lim et al. [17], with some modifications in the lateral and anterior part (Fig. 1, Supplementary video, link of the unedited video in Supplementary materials). All surgeries were performed by a single surgeon who was experienced in both procedures (*n* > 200 for conventional and Retzius-sparing approach).

#### Posterior dissection

After incision of peritoneum and isolation of vas deferens (VD) and seminal vesicles (SV), a posterior space between the prostatic capsule and Denonvilliers’ fascia (DF) was developed as previously described [16]. This plane was continued as lateral as possible.

#### Lateral dissection

Using bladder neck as the anatomical landmark, the edge of the bladder was identified. A lateral space between the prostatic fascia and endopelvic fascia was developed under the edge of bladder. This space could be continued distally between the apex and base and posteriorly to the fascial tendinous arch of pelvis (Fig. 1A).

#### Lateral pedicles control

After the development of posterior and lateral space, the lateral attachments where distributed with neurovascular bundle (NVB) was well exposed. Lateral pedicles were sharply cut after being secured by Hem-o-lock clips or bipolar cautery, from the basement towards the apex of the prostate (Fig. 1B). Different dissection planes (inter- or extra-fascial technique) could be determined depends on the never-sparing strategies, which is similar to that in the Con-RARP approach. This step was repeated on the contralateral side. Once the prostate was free posteriorly and laterally, the Retzius space was easily exposed (Fig. 1B), which was characterized by fat tissue within it. This step was also repeated on the contralateral side.

#### Dissection of the bladder neck and development of the anterior space

The bladder neck was transected and deepened posteriorly. As shown in Fig. 1C, the anterior detrusor fibers were incised, directly dissecting into the Retzius space. Of note, the anterior plane in this technique was above the detrusor apron and dorsal venous complex (DVC) to avoid the anterior positive surgical margin, which was different from any published protocols (Fig. 1C).

#### Control of dorsal venous complex

After isolation of the apex, the prostate was free posteriorly, laterally, and anteriorly, the puboprostatic ligaments were transected, and the DVC was dissected and cut without ligation. We temporarily increased abdominal CO_2_ pressure in case of DVC bleeding.

#### Dissection of urethra and anastomosis

These procedures were performed exactly according to the previously published protocols [16].

### Follow-up

All perioperative complications were filed in the medical record according to the same protocol. UC, defined as patient-reported freedom from use of pads (0 pad/day), was assessed each month for 3 months after surgery and subsequently every 3 months. Immediate UC was defined as freedom from any pad use within 7 days after removal of urinary catheter.

### Statistical analysis

All statistical analyses were performed using SPSS 22.0 statistical software (IBM SPSS, Chicago, IL, USA). Nonparametric continuous variables were presented as median and interquartile range (IQR). The Pearson Chi-square test or Fisher exact test was used to compare categorical data, while the Kruskal–Wallis test was used for comparison of continuous data between the two groups. Caliper matching was used to balance baseline information, in which caliper was set as 0.03. Equilibrium test by R language has been conducted to testify the PSM approach (supplementary material 2). Kaplan–Meier curve was presented for UC. Multivariate logistic regression analyses were applied to identify factors associated with better immediate UC recovery. A *p* < 0.05 was considered to be significant, and all p values were two-sided.

## Results

### Baseline information

Median follow-up was 60 months (IQR 50–82) for Con-RARP group and 28 months (IQR 19–39) for mRS-RARP group, respectively. Table 1 shows the baseline characteristics of the two groups. No statistically significant differences were found between the two groups regarding age, preoperative body mass index (BMI), PSA, prostate volume, American Society of Anesthesiologists (ASA) score, biopsy International Society of Urological Pathology (ISUP) group, clinical stage, and risk stratification.

**Table 1:** Baseline information of propensity score-adjusted patients with anterior tumor undergone modified Retzius-sparing or conventional RARP

	mRS-RARP	Con-RARP	% Standardized difference
Number of subjects	193	193	
Age (years), (median, IQR)	69.0 (65.0, 73.0)	69.0 (64.0, 73.0)	1.3
BMI (kg/m^2^), (median, IQR)	24.5 (22.7, 26.4)	24.4 (22.9, 26.1)	3.2
PSA (ng/dl), (median, IQR)	9.3 (6.3, 12.4)	8.8 (6.2, 13.4)	1.0
Prostate volume (ml), median (IQR)	34.7 (25.8, 46.0)	33.1 (26.8, 45.3)	0.5
ASA, *n* (%)			18.1
2	64 (33.1)	60 (31.1)	
3	127 (65.8)	133 (68.9)	
4	2 (1.0)	0 (0)	
Risk stratification, *n* (%)			16.8
1	35 (18.1)	33 (17.1)	
2	82 (42.5)	86 (44.6)	
3	76 (39.4)	74 (38.3)	
Biopsy ISUP group, *n* (%)			10.6
1	59 (30.6)	58 (30.1)	
2	55 (28.5)	60 (31.1)	
3	40 (20.7)	47 (24.4)	
4	39 (20.2)	28 (14.5)	
5	0 (0)	0(0)	
Clinical stage (MRI), *n* (%)			27.7
< T3	169 (87.6)	167 (86.5)	
T3	24 (12.4)	26 (13.5)	

*IQR* interquartile range, *BMI* body mass index, *PSA* prostate-specific antigen, *ASA* American Society of Anesthesiologists score, *ISUP* International society of urological pathology, *MRI* magnetic resonance imaging

### Perioperative and postoperative characteristics

As shown in Table 2, total operative time and console time were slightly longer for mRS-RARP (*p* = 0.006 and < 0.001, respectively). Though the estimated blood loss (EBL) in mRS-RARP group was significantly higher (200 (IQR 150,320) vs. 200 (IQR 120,240), *p* = 0.001), no differences were noted in blood transfusion rates (1.6% vs 1.6%, *p* = 1.0). All postoperative complications were grade 2 or less (such as ileus and wound pain), which were not significantly different between the two groups (4.1% vs. 2.1%, *p* = 0.241).

**Table 2:** Perioperative outcomes of propensity score-adjusted patients with anterior tumors undergone modified Retzius-sparing or conventional RARP

	mRS-RARP	Con-RARP	*p* value
Number of subjects	193	193	
Operative time, median (IQR)	172.0 (152.0, 187.0)	162.0 (142.0, 182.0)	0.006^a^
Console time, median (IQR)	111.0 (91.0, 126.0)	96.0 (76.0, 116.0)	< 0.001^a^
EBL, median (IQR)	200.0 (150.0, 320.0)	200.0 (120.0, 240.0)	0.001^a^
Blood transfusion, *n* (%)	3 (1.6)	3 (1.6)	1.0^b^
Complication, *n* (%)			0.241^b^
2 or less	8 (4.1)	4 (2.1)	
3 or greater	0 (0)	0 (0)	

*IQR* interquartile range, *EBL* estimated blood loss

^a^*p* value calculated using Kruskal–Wallis test

^b^*p* value calculated using Pearson Chi-square test or Fisher exact test

The postoperative pathological features are shown in Table 3. No differences were noted in pathological stage (*p* = 0.832) and PSMs (16.1% vs. 15.0%, *p* = 0.779).

**Table 3:** Postoperative outcomes of propensity score-adjusted patients with anterior tumors undergone modified Retzius-sparing or conventional RARP

	mRS-RARP	Con-RARP	*p* value
Number of subjects	193	193	
Postoperative ISUP, *n* (%)			0.285^b^
1	26 (13.5)	31 (16.1)	
2	97 (50.3)	108 (56.0)	
3	51 (26.4)	40 (20.7)	
4	14 (7.3)	7 (3.6)	
5	5 (2.6)	7 (3.6)	
Pathological T stage, *n* (%)			0.832^b^
T2	124 (64.2)	122 (63.2)	
T3	69 (35.7)	71 (36.8)	
PSM, *n* (%)	31 (16.1)	29 (15.0)	
pT2			0.119^b^
No	110 (88.7)	115 (94.3)	
Yes	14 (11.3)	7 (5.7)	
pT3			0.402^b^
No	52 (75.4)	49 (69.0)	
Yes	17 (24.6)	22 (31.0)	
PSM, *n* (%)	31 (16.1)	29 (15.0)	0.779^b^
Base	5 (16.1)	4 (13.8)	
Body	20 (64.5)	17 (58.6)	
Apex	11 (35.5)	13 (44.8)	
Urinary continence (immediate), *n* (%)	48 (24.9)	19 (9.8)	< 0.001^b^
Urinary continence (1 month), *n* (%)	56 (29.0)	26 (13.5)	< 0.001^b^
Urinary continence (3 months), *n* (%)	94 (48.7)	83 (43.0)	0.261^b^
Urinary continence (6 months), *n* (%)	125 (64.8)	123 (63.7)	0.832^b^
Urinary continence (12 months), *n* (%)	162 (83.9)	159 (82.4)	0.683^b^

*ISUP* International society of urological pathology, *PSM* positive surgical margin

^a^*p* value calculated using Kruskal–Wallis test

^b^*p* value calculated using Pearson Chi-square test or Fisher exact test

### Urinary continence and oncological outcomes

Kaplan–Meier curves did not show significant differences of the UC recovery 1-year post surgery (Supplementary Fig. 2). However, the UC recovery rate in mRS-RARP groups was significantly higher at catheter removal (24.9% vs. 9.8%, *p* < 0.001) and at 1 month after surgery (29.0% vs. 13.5%, *p* < 0.001). Otherwise, no significant differences were observed regarding UC in the two groups at 3-, 6-, and 12-month follow-ups (*p* = 0.261, 0.832, and 0.683, respectively) (Table 3). Multivariate logistic regression analysis indicated that modified Retzius-sparing approach was an independent factor related to better immediate UC recovery (OR 3.52, 95% CI 1.90–6.52, *p* < 0.001) (Supplementary Table 1).

## Discussion

In the present study, we described some modifications to the Retzius-sparing approach of RARP that may allow a clearer plane in the anterior part of the prostate. Compared to the conventional approach, no significant differences were found in terms of PSM rates. In addition, mRS-RARP showed an improved UC recovery at catheter removal and at 1-month after surgery. To our knowledge, we were the first group to propose modifications to the current Retzius-sparing approach aiming to balance the PSM rate and postoperative UC recovery outcomes for cases with anterior tumors. mRS-RARP showed a better early recovery of UC, without sacrificing PSM concerns.

Despite the better outcomes of UC recovery after RS-RARP, a higher PSM rate has been reported in several retrospective studies [17, 18] and prospective trials [7, 9]. Recently, pooled analyses with larger sample sizes indicated significantly higher PSMs in for RS-RARP [10, 12, 13]. The benefits of early UC recovery after RS-RARP was thought to be attributable to the preservation of many UC-related structures within the Retzius space, such as puboprostatic ligaments, DVC, and endopelvic fascia [6]. However, the plane between these structures and anterior capsule of prostate may be difficult to identify [19]. This could explain the higher PSM rate in cases with anterior tumors [14].

UC outcomes were not as good as the those reported in the previously published studies [8, 20]. This may relate to more UC-related structures in the Retzius space being resected in this modified technique, including puboprostatic ligaments, endopelvic fascia, dorsal venous complex, and detrusor apron. However, the UC rate immediate after removal of urinary catheter and at the time-point of 1-month follow-up remained higher in the near postoperative period compared to the conventional approach. This remained significant on multivariable analysis, confirming our mRS-RARP maintains a better immediate UC recovery as per the non-modified Retzius-sparing technique. This could be explained by the maintenance of bladder neck preservation, which has been demonstrated to contribute to the early UC outcomes after RS-RARP [21].

Despite some differences were observed in terms of EBL was not clinically meaningful, as shown by comparable rates of blood transfusions between the two approaches. Usually, Santorini’s plexus was ligated before the dissection of apex and urethra in the conventional approach. However, this procedure was omitted in the modified Retzius-sparing approach due to the limited anterior space, which might contribute to the more blood loss during the mRS-RARP. However, to our experience, the blood loss in mRS-RARP was controllable. We temporarily increased abdominal CO_2_ pressure in case of DVC bleeding. In addition, DVC was rarely sutured in mRS-RARP since this space was pressed after vesicourethral anastomosis.

Our study was not without limitations. First, it is a retrospective work. Nonetheless, we performed a matched pair comparison to decrease possible group imbalances. Second, mRS-RARP was performed later in the surgeon learning curve which may introduce experience-related bias. Nonetheless, the surgeon was experienced in both procedures. In addition, the long-term oncological outcomes, such as BCR-free survival, were not available for analysis due to the limited follow-up duration of mRS-RARP.

In conclusion, we described a modified Retzius-sparing approach of RARP in patients with anterior tumors. This technique might allow earlier continence recovery while maintaining similar positive surgical margin rates in comparison to conventional RARP. Further prospective studies were needed to evaluate the generalizability and reproducibility of this technique in terms of oncological and functional efficiency.

## Supplementary Information

Below is the link to the electronic supplementary material.

Supplementary file1 (DOCX 94 KB)

Supplementary file2 (MP4 291,432 KB)

## Data Availability

Data available on request from the authors.
