# Supplementary material for: Modified Retzius-sparing robot-assisted radical prostatectomy for cases with anterior tumor: a propensity score-matched analysis
Source: World J Urol. 2024 Mar 20;42(1):170. doi: 10.1007/s00345-024-04807-7 (PMC10954873; doi:10.1007/s00345-024-04807-7)
Supplement: Supplementary file 1 — Supplementary file1 (DOCX 94 KB) [file 345_2024_4807_MOESM1_ESM.docx]

**
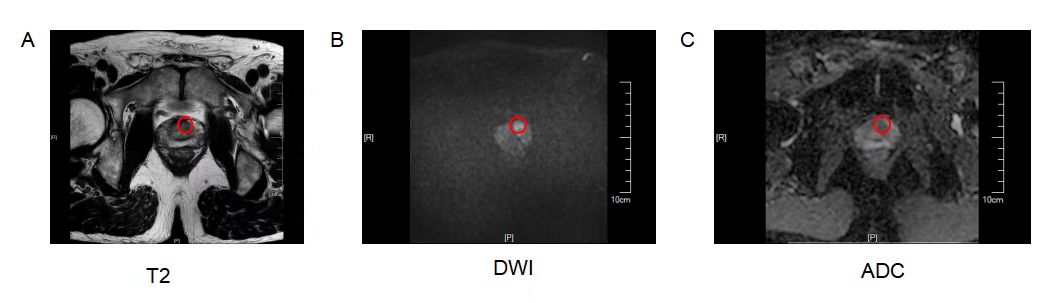
**

**Supplementary Figure 1: A representative case with anterior tumor included in the represent study. An anterior lesion (PI-RADS 4) was identified on the pre-operative MRI images (A-C).**


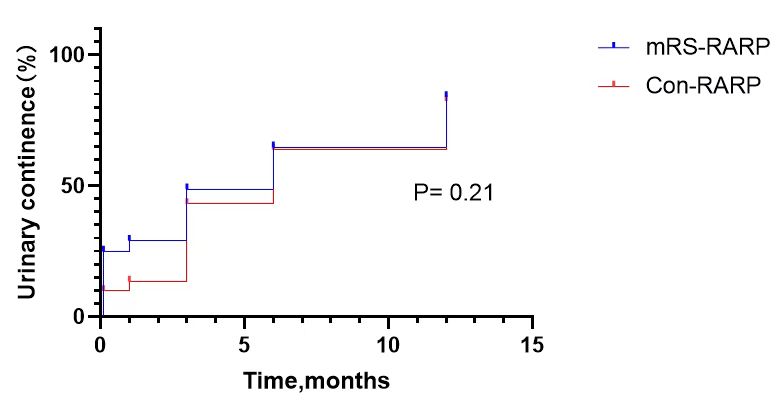


**Supplementary Figure 2: Kaplan-Meier Curve of the urinary continence recovery in patients underwent modified Retzius-sparing or conventional robot-assisted radical prostatectomy.**

**Supplementary Table 1: Multivariable competing risks regression of propensity score-adjusted patients underwent modified Retzius-sparing or conventional robot-assisted radical prostatectomy.**

| Variables mRS-RARP versus conventional RARP | | |
| --- | --- | --- |
|  | OR(95%CI) | p value |
| Age | 0.92 (0.88-0.96) | <0.001 |
| BMI | 0.97 (0.87-1.09) | 0.644 |
| PSA | 1.0 (0.96-1.04) | 0.857 |
| Prostate volume | 0.98 (0.96-1.0) | 0.089 |
| Approach | 3.52 (1.90-6.52) | <0.001 |
| ASA |  |  |
| 2 | Reference |  |
| 3 | 0.79 (0.42-1.47) | 0.456 |
| 4 | NA | NA |
| Risk stratification |  |  |
| 1 | Reference |  |
| 2 | 0.27 (0.08-0.92) | 0.037 |
| 3 | 0.11 (0.02-0.45) | 0.003 |
| Biopsy ISUP group |  |  |
| 1 | Reference |  |
| 2 | 2.11 (0.63-7.05) | 0.226 |
| 3 | 2.41 (0.71-8.25) | 0.161 |
| 4 | 1.40 (0.30-6.65) | 0.67 |
| Clinical stage | 0.95 (0.23-3.92) | 0.944 |

BMI= body mass index, PSA= prostate specific antigen, ASA= American Society of Anesthesiologists score, ISUP = International society of urological pathology

**Supplementary Table 2: Equilibrium test of propensity score-adjusted patients underwent modified Retzius-sparing or conventional robot-assisted radical prostatectomy**


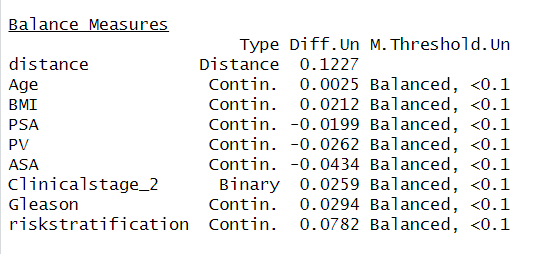


**Link to the unedited version of the video:** **https://youtu.be/Z8eDsDX50Xw**
